# Modification of Electrospun CeO_2_ Nanofibers with CuCrO_2_ Particles Applied to Hydrogen Harvest from Steam Reforming of Methanol

**DOI:** 10.3390/ma15248770

**Published:** 2022-12-08

**Authors:** Kai-Chun Hsu, Chung-Lun Yu, Heng-Jyun Lei, Subramanian Sakthinathan, Po-Chou Chen, Chia-Cheng Lin, Te-Wei Chiu, Karuppiah Nagaraj, Liangdong Fan, Yi-Hsuan Lee

**Affiliations:** 1 Department of Materials and Mineral Resources Engineering, National Taipei University of Technology, No. 1, Section 3, Zhongxiao East Road, Taipei 106, Taiwan; 2 Institute of Materials Science and Engineering, National Taipei University of Technology, No. 1, Section 3, Chung-Hsiao East Road, Taipei 106, Taiwan; 3 Graduate Institute of Organic and Polymeric Materials, National Taipei University of Technology, No. 1, Section 3, Zhongxiao East Road, Taipei 106, Taiwan; 4 E-Current Co., Ltd., 10F.-5, 50, Section 4, Nanjing East Road, Taipei 10533, Taiwan; 5 SRICT-Institute of Science and Research, UPL University of Sustainable Technology, Vataria, Ankleshwar 393135, Gujarat, India; 6 Department of New Energy Science and Technology, College of Chemistry and Environmental Engineering, Shenzhen University, Shenzhen 518060, China; 7 Department of Mechanical Engineering, National Taipei University of Technology, No. 1, Section 3, Zhongxiao East Road, Taipei 106, Taiwan

**Keywords:** delafossite, CuCrO_2_-CeO_2_, electrospinning, catalyst, methanol, hydrogen production

## Abstract

Hydrogen is the alternative renewable energy source for addressing the energy crisis, global warming, and climate change. Hydrogen is mostly obtained in the industrial process by steam reforming of natural gas. In the present work, CuCrO_2_ particles were attached to the surfaces of electrospun CeO_2_ nanofibers to form CeO_2_-CuCrO_2_ nanofibers. However, the CuCrO_2_ particles did not readily adhere to the surfaces of the CeO_2_ nanofibers, so a trace amount of SiO_2_ was added to the surfaces to make them hydrophilic. After the SiO_2_ modification, the CeO_2_ nanofibers were immersed in Cu-Cr-O precursor and annealed in a vacuum atmosphere to form CeO_2_-CuCrO_2_ nanofibers. The CuCrO_2_, CeO_2_, and CeO_2_-CuCrO_2_ nanofibers were examined by X-ray diffraction analysis, transmission electron microscopy, field emission scanning electron microscopy, scanning transmission electron microscope, thermogravimetric analysis, and Brunauer–Emmett–Teller studies (BET). The BET surface area of the CeO_2_-CuCrO_2_ nanofibers was 15.06 m^2^/g. The CeO_2_-CuCrO_2_ nanofibers exhibited hydrogen generation rates of up to 1335.16 mL min^−1^ g-cat^−1^ at 773 K. Furthermore, the CeO_2_-CuCrO_2_ nanofibers produced more hydrogen at lower temperatures. The hydrogen generation performance of these CeO_2_-CuCrO_2_ nanofibers could be of great importance in industry and have an economic impact.

## 1. Introduction

Hydrogen is a viable renewable energy source for addressing the threat of global warming and the decline of fossil fuels. Fuel cells are one of the latest technologies that may effectively convert chemicals into electrical energy to reduce these pollutants [1,2]. Proton-exchange membrane fuel cells (PEMFCs) in particular are systems with zero pollution emissions because they convert chemical energy into electrical energy during the electrochemical reaction of hydrogen and oxygen. Because the anodic Pt-based catalyst can only take less than 10 ppm of CO, PEMFCs typically require a supply of high-purity hydrogen [3,4]. Several methods can be used to produce hydrogen, but the main one is the steam reforming of natural gas. However, the use of hydrogen poses challenges in terms of production and storage [5,6]. The use of steam reforming of methanol (SRM) to produce hydrogen will effectively solve the above problems. SRM has attracted attention because of its low-temperature need for reaction, water-solubility, endothermic process, and high hydrogen yields, which make it ideal for fuel cell applications [7,8]. Decomposition, steam reforming, and partial oxidation are the three primary methods utilized to produce hydrogen from CH_3_OH [9,10].

CH_3_OH → CO + 2H_2_………………………….... ΔH^0^ = 128 kJ/mol (1)

CH_3_OH + H_2_O → CO_2_ + 3H_2_……………………ΔH^0^ = 131 kJ/mol (2)

CH_3_OH + ½O_2_ → CO_2_ + 2H_2_……………………ΔH^0^ = 155 kJ/mol (3)

At a high CO ratio, the decomposition process is an extremely endothermic breakdown process. As a result, this method is ineffective for fuel cells [11]. The partial oxidation reaction process is a highly exothermic reaction with 66% hydrogen output. This process uses pure oxygen instead of air. Finally, the endothermic steam reforming process produces a high rate of hydrogen, up to 75% on a dry basis, with CO as a byproduct. As a result, the steam reforming process is more advantageous for hydrogen production [12,13]. These traditional methods for converting SRM into hydrogen comprise processes like CH_3_OH decomposition, water gas shift, and the SRM process [14].

The SRM process is appropriate for producing hydrogen due to its low reaction temperature, adequate water miscibility, high hydrogen concentration ratio, and low CO level. It is also a direct and cost-effective method of hydrogen production. In addition, it is endothermic and can produce a large amount of hydrogen, which is favorable for fuel cell usage [15,16].

The performance of steam re-forming at the reactor is highly impacted by the reaction conditions and catalyst preparation. In the SRM reaction, Ru, Zn, Pd, Ni, Cu, and a combination of these metal-based catalysts are frequently used. Cu-based catalysts are particularly suitable for hydrogen production in the SRM process. Copper and copper-based catalyst materials have a high operating temperature of 573 K, although deactivation occurs at 573–623 K due to the thermal frittage of Cu particles. A further key issue with the SRM process is the deactivation of the catalyst due to the deposition of carbon particles on the Cu catalyst surface [17,18].

To alleviate these issues, a metallic oxide such as ZrO_2_, Fe_2_O, ZnO, and CeO_2_ can be combined with copper and copper-based catalysts to enhance the catalytic performance. With the metallic oxide, the enhanced Cu catalyst will have a fine dispersion with high efficiency and thermal stability. Catalysts have a big impact on the formation of hydrogen in the SRM reaction and the final products. Hence, metal oxide catalysts like Al_2_O_3_, ZnO/Al_2_O_3_, ZrO_2_/Al_2_O, Cr_2_O_3_/Al_2_O_3_, CuO/ZnO/Al_2_O_3_, and CeO_2_/ZrO_2_ are used as a catalyst for the SRM reaction [19,20]. However, the primary issue with the SRM process is the deposition of carbon particles on the surface of the Cu-related catalyst, which lowers the catalyst’s effectiveness. To improve the efficiency of hydrogen production, many studies have focused on the use of delafossite materials in the SRM process [21,22].

The chemical formula of delafossite is ABO_2_, where A is a cation with linear coordination to two oxygen ions that are often occupied by a cation of a noble metal with a univalent oxidation state, such as Pt^1+^, Cu^1+^, or Ag^1+^. The central metal of the distorted edge-shared BO_6_ octahedron is cation B, which has a trivalent charge, such as B^3+^, Al^3+^, Ga^3+^, Cr^3+^, or Fe^3+^. Delafossite is a translucent conductive oxide applied in optoelectronic technology [23,24,25]. However, research regarding its application to catalysis has been scant. Previous studies have applied it to methanol synthesis, N_2_O decomposition [26], methanol steam reforming [27], HCl oxidation [28], photocatalytic hydrogen processing, and NO_3_ elimination, among other things [29].

Cerium oxide has numerous applications, some being catalysis, ceramics, gas sensors, fuel cell, biomaterials, and solid electrolytes [30]. The most important characteristic of CeO_2_ is that it transfers oxygen via the redox potential transfer between Ce^4+^ and Ce^3+^ under oxidation and reduction conditions [31]. Li et al. have reported that adding CeO_2_ can reduce the catalytic temperature of Cu-based catalysts and promote catalytic efficiency [32]. Electrospinning was first patented in the United States in 1902 by John Francis Cooley [33]. This method can produce one-dimensional fibers in the micrometer to nanometer diameter ranges with large active surface areas and high porosity. Oxide nanofibers have already been used in energy and environmental applications, such as sensors, catalysis, biotechnology, solar cells, hydrogen energy, and super-capacitors [34].

The CeO_2_-CuCrO_2_ nanofiber catalyst was synthesized by the self-combustion glycine–nitrate process (GNP) and applied for SRM in this study. The CeO_2_-CuCrO_2_ nanofibers had a nanosized, spherical shape with a crystalline delafossite structure. Furthermore, the hydrogen production rate of CeO_2_-CuCrO_2_ nanofibers was compared with those of CuCrO_2_, CeO_2_, and commercial Cu/Al/Zn catalysts. Based on the comparison, the CeO_2_-CuCrO_2_ nanofibers exhibited higher hydrogen yields with lower coke formation during the SRM process as compared with CuCrO_2_, CeO_2_, and commercial Cu/Al/Zn catalysts.

## 2. Materials and Methods

### 2.1. Instrumentation

The starting reagents, namely copper nitrate hexahydrate ([Cu(NO_3_)_3_·6H_2_O]), chromium nitrate nonahydrate ([Cr(NO_3_)_3_·9H_2_O]), cerium nitrate hexahydrate ([Ce(NO_3_)_3_·6H_2_O]), N, N-dimethylformamide, Triton X100 polyvinylpyrrolidone (PVP) (M. W = 1,300,000 g·mol^−1^), and tetraethyl orthosilicate (TEOS), were obtained from SHOWA and Sigma-Aldrich. In this study, the CeO_2_ nanofibers and CeO_2_-CuCrO_2_ nanofibers were examined using the appropriate instrumentation techniques. By using an X-ray diffractometer (D2 Phaser, Bruker) with a working voltage of 30 kV and CuK radiation, the crystalline structures of the nanofibers were examined. Field emission scanning electron microscopy (FESEM) (Regulus-8100, HITACHI, Tokyo, Japan) and transmission electron microscopy studies (FE-2100TEM, JEOL, Tokyo, Japan) were used in this work to examine the morphology and particle size of the catalyst. A thermogravimetric analysis/differential scanning calorimeter (TGA/DSC, STA 449 F5, NETZSCH, Selb, Germany) was used to investigate the thermal degradation behavior of electrospun fibers. The specific surface area was calculated using the Brunauer–Emmett–Teller (BET) method using a Micromeritics TriStar II 3030 specification. Before a BET measurement was performed, an appropriate quantity of the produced catalyst was de-gassed at 473 K for 24 h to eliminate the absorbed water. At different relative pressures (P/P_0_) ranging from 0 to 0.3, N_2_ adsorption isotherms were observed and examined while the catalyst absorbed N_2_.

### 2.2. Preparation of CeO_2_ Nanofibers

The precursor solution was synthesized by dissolving 0.625 g of cerium nitrate in 14.4 mL of N, N-dimethylformamide. Following that, 2.4 g of PVP was dissolved in the above precursor solution. After 6 h of stirring, a bright yellow viscous gel-like reaction precursor solution was obtained. This solution was electrospun with a working distance of 15 cm, a voltage of 18 kV, a flow rate of 0.02 mL/h, temperature controlled at 313 K, and humidity of less than 20%. The as-spun fiber was annealed at 873 K with a heating rate of 274 K per minute, and the resulting CeO_2_ nanofibers were analyzed by XRD and SEM studies.

### 2.3. Surface Modification of CeO_2_ Nanofibers

The surfaces of the CeO_2_ nanofibers were modified as follows. Because CuCrO_2_ would not easily adhere directly to the surfaces of the CeO_2_ nanofibers, the surfaces were coated with SiO_2_. Hence, the CeO_2_ fibers were dipped into tetraethyl orthosilicate (TEOS) and then annealed in air at 873 K. The modified CeO_2_ nanofibers were analyzed by XRD, SEM, and TEM studies.

### 2.4. Preparation of CeO_2_ Nanofibers Decorated with CuCrO_2_ Nanoparticles (CeO_2_-CuCrO_2_)

CeO_2_ nanofibers decorated with CuCrO_2_ nanoparticles (CeO_2_-CuCrO_2_) were prepared with the following procedure. The CeO_2_ fibers were dipped in the precursor, which was a mixture of methanol, chromium nitrate, copper nitrate, and Triton X100, and then dried at 353 K for 2 min before being annealed at 1073 K in a vacuum with a heating rate of 283 K per minute. The prepared CeO_2_-CuCrO_2_ nanofibers were analyzed by XRD, SEM, and TEM.

### 2.5. Steam Reforming of Methanol Process over Electrospun CeO_2_-CuCrO_2_ Nanofibers Catalyst

**Figure 1:**
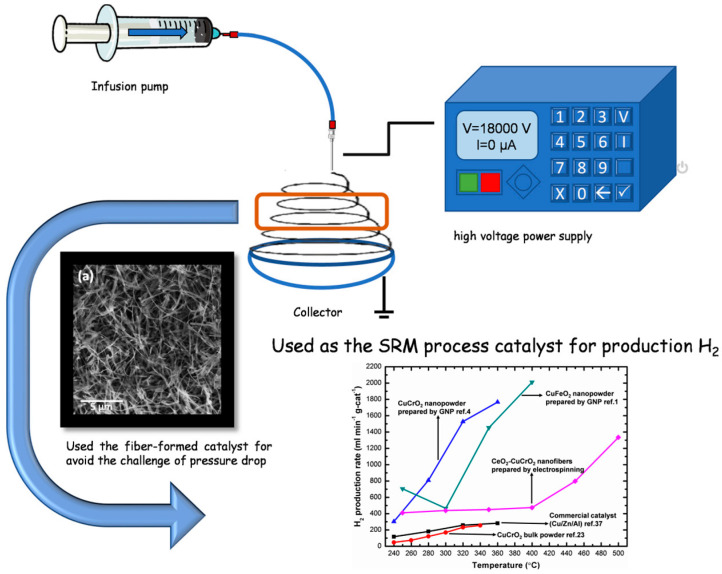
Schematic diagram of the methanol steam reforming process on the electrospun CeO_2_-CuCrO_2_ nanofibers catalyst.

The steam reforming of methanol was performed in a tubular flow reactor using a 25 cm quartz tube with a 1.2 cm inner diameter, nitrogen as the carrier gas with a flow rate of 30 sccm, and 20 mg of catalyst per SRM reaction. The system was connected to a gas chromatograph for analysis. A methanol–water mixture was prepared in a 3:1 molar ratio and heated to 353 K on a hot plate to evaporate methanol–water vapor. A gas tube was inserted into the Erlenmeyer flask beneath the level of the methanol aqueous solution, and then the methanol vapor was carried by nitrogen to the catalyst for the reaction. The nanofibers were sandwiched between quartz cotton in the middle of the quartz tube and then heated to 523, 573, 623, 673, 723, and 773 K, respectively. A gas chromatograph (GC 1000 China Chromatography TCD) was used to analyze each temperature and identify the average values (Figure 1).

## 3. Results and Discussion

### 3.1. XRD Analysis

**Figure 2:**
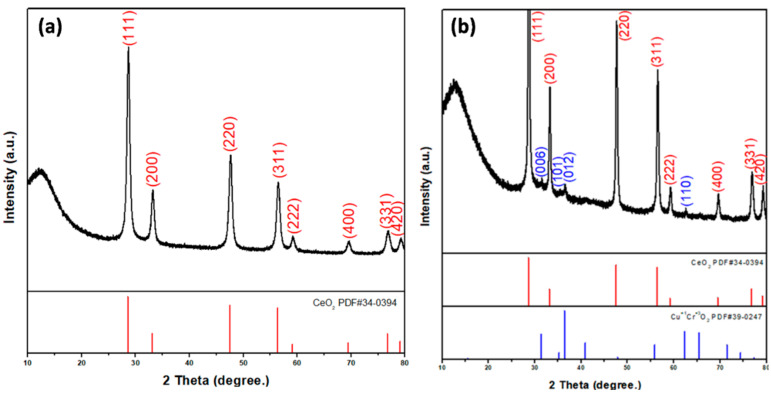
XRD patterns of (**a**) CeO_2_ and (**b**) CeO_2_-CuCrO_2_ nanofibers.

The prepared nanofiber diffraction patterns and crystal structures were studied by XRD studies and analyzed with powder X-ray diffractometric MDI JADE5.0 software tools. Figure 2a presents the XRD pattern of CeO_2_ nanofibers, showing the pure CeO_2_ cubic phase (JCPDS card PDF#34-0394.) After the electrospun CeO_2_ nanofibers were annealed at high temperature, the cerium nitrate decomposed and CeO_2_ remained.

Figure 2b shows the XRD pattern of CuCrO_2_-coated CeO_2_ nanofibers. The XRD spectra of CeO_2_-CuCrO_2_ nanofibers exhibited that CuCrO_2_ nanoparticles were attached to the CeO_2_ nanofibers. The XRD pattern reveals CeO_2_ (PDF#34-0394) and CuCrO_2_ (PDF#39-0247) phases on the CeO_2_-CuCrO_2_ nanofibers. The XRD pattern of CeO_2_ nanofibers after SiO_2_ surface modification only reveals CeO_2_ (PDF#34-0394) due to annealing at 873 K and its SiO_2_ content being too low.

**Figure 3:**
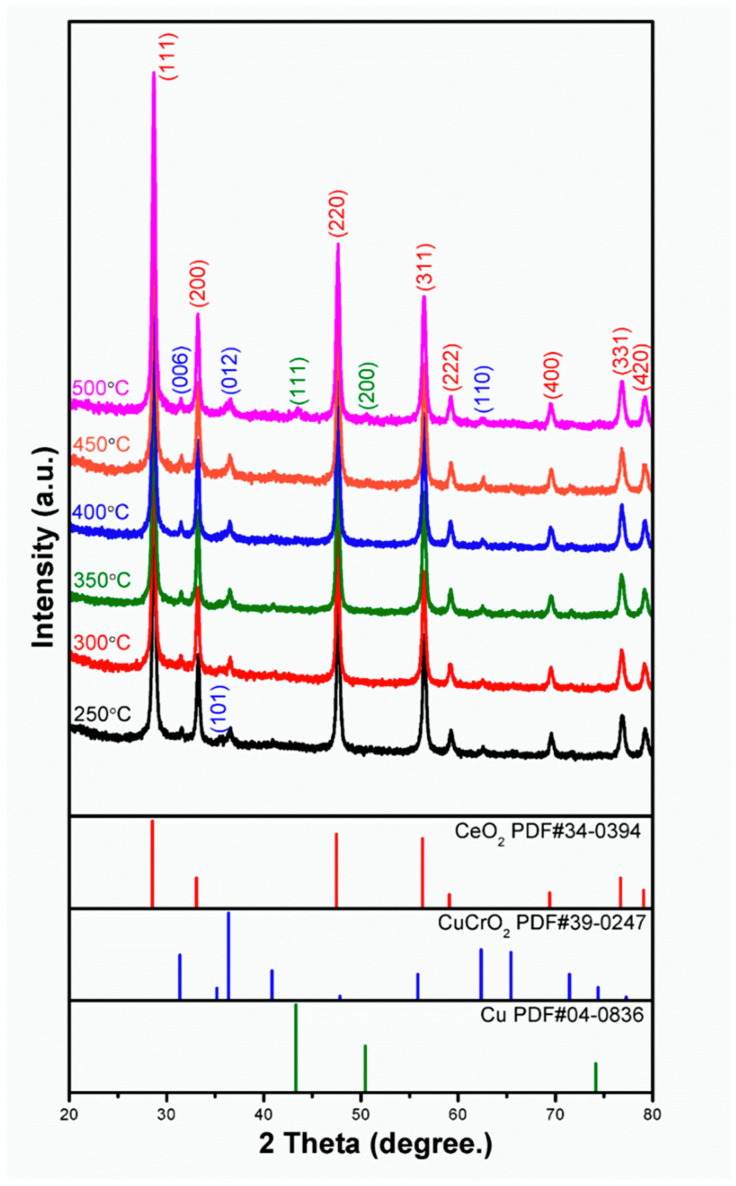
XRD pattern of CeO_2_-CuCrO_2_ nanofibers after SRM at different temperatures.

Figure 3 shows the XRD pattern of CeO_2_-CuCrO_2_ after SRM at different temperatures. From the XRD pattern, it can be observed that when the catalytic temperature increases, the peak of CuCrO_2_(101) at 2θ = 35.178°(PDF#39-0247) gradually disappears. At 773 K, due to the precipitation of CuCrO_2_ after catalysis, the peaks of the copper (111) and (200) planes can be observed at 2θ = 43.297° and 50.433° (PDF#04-0836), respectively.

### 3.2. FESEM Analysis

**Figure 4:**
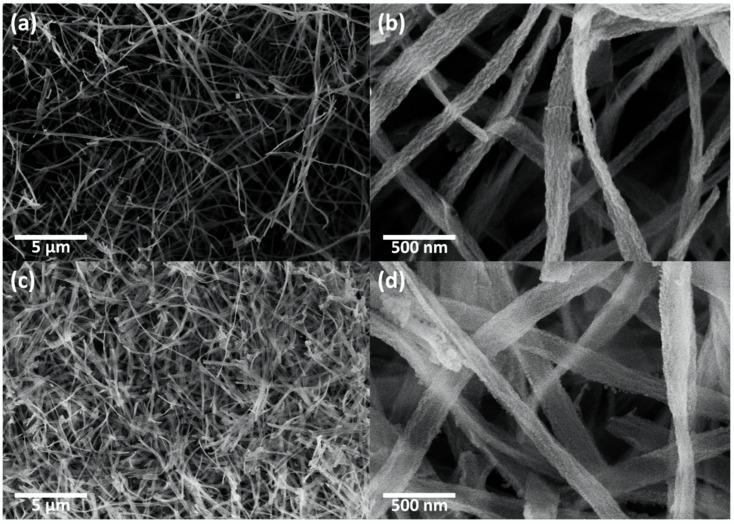
FESEM image of (**a**,**b**) CeO_2_ nanofibers and (**c**,d) CeO_2_-CuCrO_2_ nanofibers.

The morphologies of the CeO_2_ nanofibers and CeO_2_-CuCrO_2_ nanofibers were identified by FESEM and TEM studies. Figure 4 shows the FESEM image of electrospun CeO_2_ and CeO_2_-CuCrO_2_ nanofibers. Figure 4a,b show the CeO_2_ nanofibers after annealing at a rate of 274 K/min to 873 K in an air atmosphere. The CeO_2_ nanofibers decreased in size by about 110 nm and disappear from PVP due to annealing. Figure 4c,d show FESEM images of CeO_2_-CuCrO_2_ nanofibers. It was found that CuCrO_2_ is difficult to directly attach to the surface of CeO_2_; therefore, the surface was modified with SiO_2_ to improve the CuCrO_2_ adherence. The FESEM images show that the CeO_2_-CuCrO_2_ nanofibers were very thin, with diameters similar to those of the CeO_2_ nanofibers.

**Figure 5:**
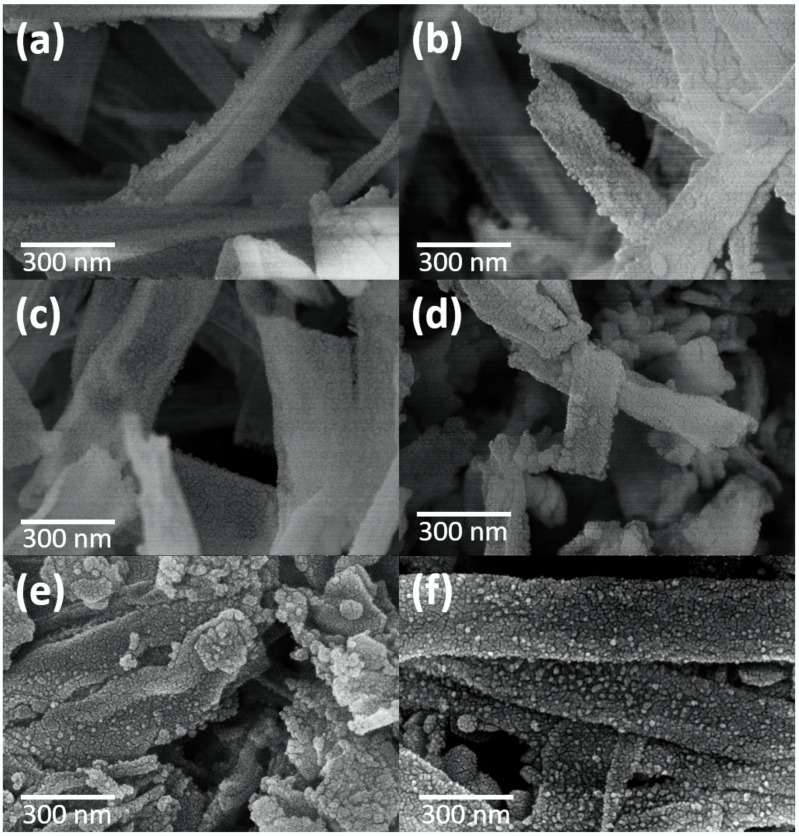
FESEM images of CeO_2_-CuCrO_2_ nanofibers after SRM at different temperatures (**a**) 523 K, (**b**) 573 K, (**c**) 623 K, (**d**) 673 K, (**e**) 723 K, and (**f**) 773 K.

Figure 5 shows the FESEM images of CeO_2_-CuCrO_2_ after SRM at different temperatures. The morphologies of CeO_2_-CuCrO_2_ nanofibers after SRM at 523–623 K exhibited the fiber structure, revealing that the CeO_2_-CuCrO_2_ nanofibers had better stability at lower reaction temperatures, as can be seen in Figure 5a–c. Figure 5d shows that the morphology of the nanofibers becomes more fragmented when the catalytic temperature reaches 673 K. From Figure 5e,f, it can be observed that when the catalytic temperature reaches 723 K, copper-precipitated particles begin to appear on the surface of the nanofibers. When the catalytic temperature reaches 773 K, the precipitated particles are scattered on the surface.

### 3.3. TEM and STEM-EDS Analysis

**Figure 6:**
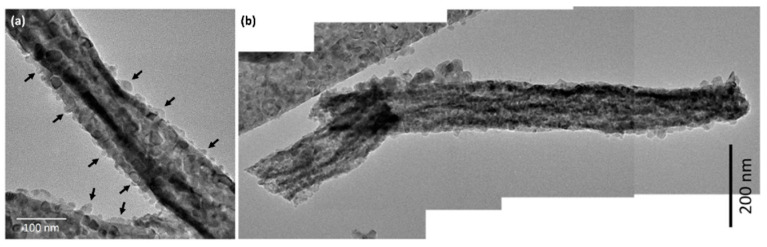
(**a**,**b**) TEM image of CeO_2_-CuCrO_2_ nanofiber.

**Figure 7:**
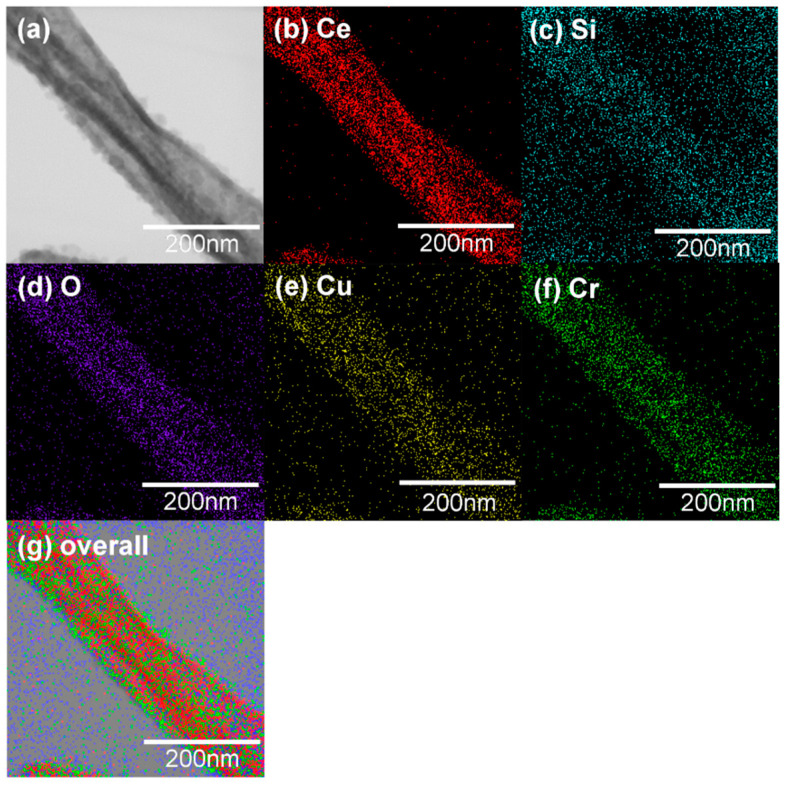
(**a**) STEM image of CeO_2_-CuCrO_2_ nanofiber, (**b**) Ce, (**c**) Si, (**d**) O, (**e**) Cu, (**f**) Cr. (**g**) Overall STEM-EDS mapping of Ce, Si, O, Cu and Cr present in CeO_2_-CuCrO_2_ nanofiber.

**Figure 8:**
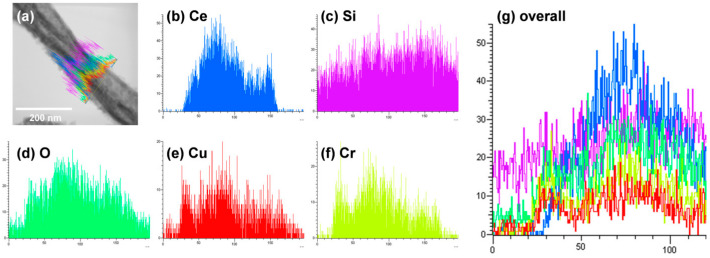
STEM image of CeO_2_-CuCrO_2_ nanofibers. (**a**) Overall STEM spectrum of Ce, O, Si, Cu, and Cr overlap; Elemental mapping of (**b**) Ce, (**c**) Si, (**d**) O, (**e**) Cu, (**f**) Cr. (**g**) Overall STEM-EDX spectrum of Ce, O, Si, Cu, and Cr overlap.

Figure 6a,b shows TEM images of CeO_2_-CuCrO_2_ nanofibers. These images confirmed that the CuCrO_2_ particles were arranged and attached to the surface of CeO_2_ nanofibers. The STEM image in Figure 7a confirmed that the CeO_2_ nanofibers had particles arranged on the surface and attached to them. The particle attached to the fiber were investigated by STEM-EDS mapping. The STEM-EDS mapping confirmed the presence of (b) Ce, (c) Si, (d) O, (e) Cu, and (f) Cr in the CeO_2_-CuCrO_2_ nanofibers. Figure 7g shows the overall STEM-EDS mapping, which confirmed the presence of Ce, Si, O, Cu, and Cr in the CeO_2_-CuCrO_2_ nanofibers. Figure 8 shows the STEM-EDS results of modified CeO_2_ nanofibers coated with CuCrO_2_. Figure 8a confirms the presence of Ce, Si, O, Cu, and Cr in the CeO_2_-CuCrO_2_ nanofibers. The STEM-EDS spectra of CeO_2_-CuCrO_2_ nanofibers after SiO_2_ surface modification show that (b) Ce, (c) Si, (d) O, (e) Cu, and (f) Cr were present in the CeO_2_-CuCrO_2_ nanofibers. Figure 8g shows the STEM-EDS overall spectra confirming that the layer on the fiber surface was mainly composed of CeO_2_-CuCrO_2_. Hence, TEM and STEM-EDS studies confirmed the formation of CeO_2_-CuCrO_2_ nanofibers.

### 3.4. TGA Analysis

**Figure 9:**
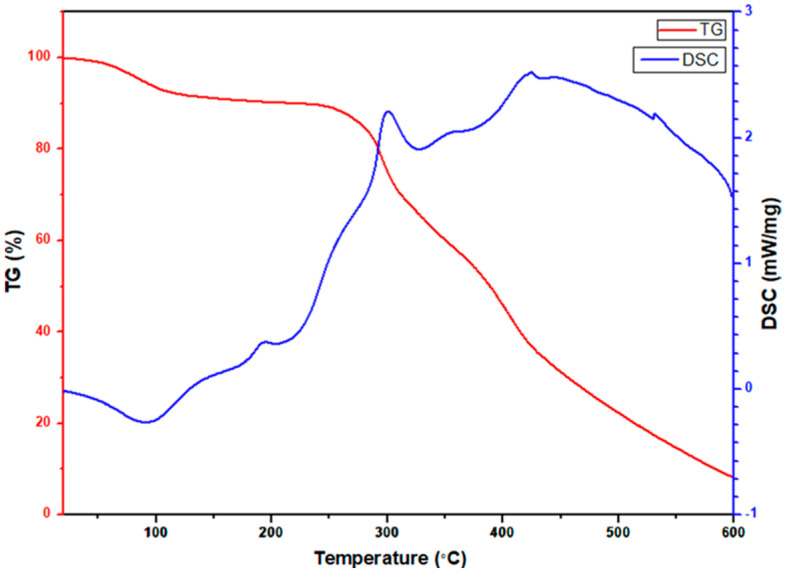
TGA/DSC studies of electrospun CeO_2_-CuCrO_2_ nanofibers.

To observe the decomposition mechanism of the as-spun nanofibers at high temperatures, a simultaneous thermogravimetric analyzer was used to observe the TGA/DSC curve, and the temperature was increased to 873 K at a rate of 283 K per minute in air. Figure 9 shows the TGA/DSC curve of electrospun CeO_2_-CuCrO_2_ fibers. According to the TGA/DSC studies, the weight loss before 373 K is due to the volatilization of the remaining water in the CeO_2_-CuCrO_2_ fibers. The slight weight loss at approximately 423 K and the exothermic slope are due to DMF decomposition, and the endothermic peak at around 493 K is due to cerium nitrate decomposition. After that, a massive and continuous weight loss at about 523 K indicates the significant decomposition of PVP. Moreover, at 573 K to 673 K, an endothermic peak signals the formation of CeO_2_-CuCrO_2_ nanofibers.

### 3.5. Specific Surface Area Analysis

The specific surface area of the CeO_2_-CuCrO_2_ nanofibers is listed in Table 1. Table 1 shows the BET-specific surface areas of delafossite materials produced by solid-state reaction, glycine combustion, and electrospinning. The specific surface area of the CeO_2_-CuCrO_2_ nanofibers produced in this experiment is 15.06 m^2^/g, which is larger than the specific surface area of solid-state reactions and other electrospun products.

### 3.6. Steam Reforming of Methanol Performance

**Figure 10:**
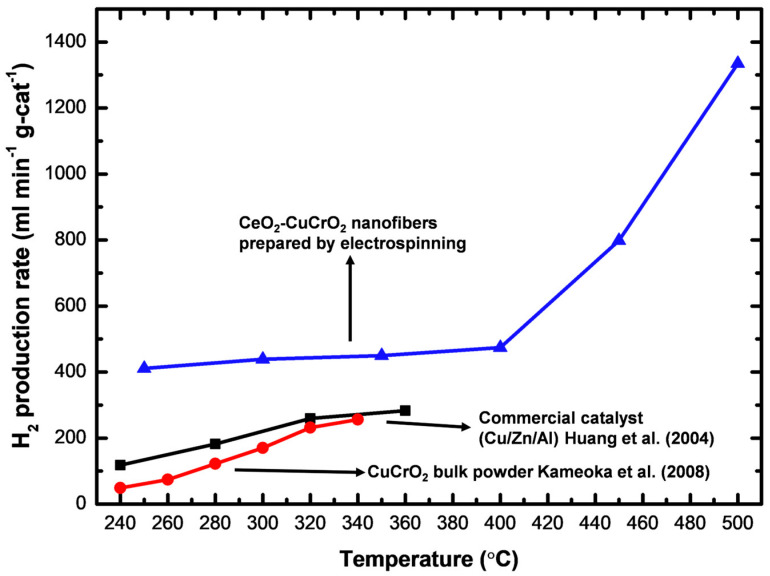
Hydrogen production of electrospinning prepared CeO_2_-CuCrO_2_, compared with CuCrO_2_ (solid-state method) [24] and commercial Cu/Zn/Al catalysts [35].

A gas chromatograph was attached to the thermal conductivity detector and used for measuring the rate of hydrogen production. At a flow velocity of 30 sccm and temperatures between 523 K and 773 K, the hydrogen generation was measured using 0.04 g of catalyst, and the hydrogen production rate was converted into mL min^−1^ g-cat^−1^. To obtain high catalytic performance, the prepared CeO_2_-CuCrO_2_ catalyst was heated without contact with methanol vapor. Additionally, the carrier gas was altered so that the system was filled with methanol steam, and the gas coming from the exit tube was located. As shown in Table 2, the SRM process was carried out over the CeO_2_-CuCrO_2_ catalysts at 523–673 K at a flow rate of 30 sccm. Additionally, when the reaction temperature was raised from 523–673 K, the rate of hydrogen generation increased. The results are shown in Figure 10. In Figure 10, the CeO_2_-CuCrO_2_ nanofiber exhibited an excellent hydrogen production performance compared with CuCrO_2_ (solid-state method) and commercial Cu/Zn/Al catalysts [24,35]. Table 2 shows the hydrogen production rate of CeO_2_-CuCrO_2_ nanofibers at different temperatures—the hydrogen production rises with an increase in temperature. However, the CeO_2_-CuCrO_2_ nanofibers lose their activity at the reaction temperature is too high, therefore the experiment has not been continued to a high temperature. The CeO_2_-CuCrO_2_ nanofibers are extremely stable in air, in contrast to an H_2_-activated catalyst, which is typically harmful when exposed to air due to its high activity and potential for ignition and explosion. Therefore, there is no need to activate the CeO_2_-CuCrO_2_ nanofiber catalyst at high temperatures for the SRM process. This study implies that greater efficiency can be attained than with traditional catalysts if CeO_2_-CuCrO_2_ nanofibers are installed in a fuel cell vehicle. Future research will examine the stability of the catalyst, SRM conditions, and reactor condition optimization.

## 4. Conclusions

In this study, the electrospun CeO_2_-CuCrO_2_ nanofiber catalyst was effectively created and used for steam reforming of methanol (SRM). The prepared nanofiber catalyst was evaluated by the field emission scanning electron microscope, transmission electron microscope, X-ray diffractometer energy-dispersive X-ray spectroscopy, thermogravimetric analyzer (STA), and Brunauer-Emmett-Teller analysis. The specific surface area of the CeO_2_-CuCrO_2_ nanofibers is 15.06 m^2^/g. The best hydrogen production rate of the CeO_2_-CuCrO_2_ nanofibers, 1335.16 mL min^−1^ g-cat^−1^, was achieved at a flow rate of 30 sccm and reaction temperature of 773 K. Furthermore, the optimization of reduction conditions and catalyst stability were studied. According to the findings, the increased hydrogen production rate can be ascribed to the stronger catalytic activity, larger surface area, lower reactor temperature, and higher methanol flow rate of the CeO_2_-CuCrO_2_ nanofiber catalyst. According to the H_2_ production performance, the CeO_2_-CuCrO_2_ nanofibers can be employed as a better catalyst for commercial H_2_ production and are suited for fuel cell vehicles without high-temperature activation.

## Figures and Tables

**Table 1:** Specific surface area of the different delafossite materials prepared by various processes.

Processes	Specific Surface Area (m^2^/g)	Reference
GNP method (CuFeO_2)_	11.38	[16]
Solid-state (CuCrO_2_)	1.94	[17]
Solid-state (CuFeO_2_)	0.57	[17]
Electrospinning (CuCrO_2_)	7.85	[18]
Electrospinning (CuFeO_2_)	4.33	[19]
Electrospinning (CeO_2_-CuCrO_2_)	15.06	This study

**Table 2:** Hydrogen production rate of CeO_2_-CuCrO_2_ nanofibers at different temperatures.

Temperature (Kelvin)	H_2_ Production Rate (mL min^−1^ g-cat^−1^)
523	410.66
573	438.48
623	451.52
673	474.30
723	798.28
773	1335.16

## Data Availability

Not applicable.
